# A Retrospective Study Comparing Nose, Lip, and Chin Morphology in Class I, Class II, and Class III Skeletal Relationships in Patients Visiting to the Department of Orthodontics, BPKIHS: A Cephalometric Study

**DOI:** 10.1155/2022/2252746

**Published:** 2022-08-22

**Authors:** Avinash Chaudhary, Jamal Giri, Rajesh Gyawali, Prabhat Ranjan Pokharel

**Affiliations:** Department of Orthodontics, CODS, BPKIHS, Dharan, Nepal

## Abstract

**Background:**

With the changing paradigm from primarily hard tissue to soft tissue evaluation for orthodontic diagnosis and treatment planning, the priority has shifted to bring about improvement in the profile and smile characteristics of patients. Since not only the esthetics but also the stability of orthodontic treatment is largely determined by the soft tissue envelope, proper positioning of the soft tissue drape becomes paramount. Soft tissues of face, namely, nose, lips, and chin, are of paramount importance not only from an esthetic but also from functional and treatment stability points.

**Objective:**

To determine the morphological variation of lips, nose, and chin in different skeletal malocclusions.

**Materials and Methods:**

Lateral cephalograms of 237 patients visiting the department of orthodontics, BPKIHS, were taken, hand traced on matt acetate tracing paper of 0.002″ thickness with 0.3-mm 2B pencil. Samples were divided into 3 skeletal classes based on ANB angle. Measurements were made in relation to the nose, lips, and chin. Data were inserted in to SPSS and analyzed statistically using descriptive statistics, and mean and standard deviation was calculated for each variable. Multiple comparison between groups was done with post hoc Bonferroni test with mean difference significant at *p* < 0.05.

**Result:**

On intergroup comparison, a significant difference was found for upper lip thickness (ULT) between Class II and Class III, and lower lip length (LLL) between Class I and Class III, and between Class II and Class III. Significant difference for nasolabial angle (NLA) was found between Class II and Class III. Similarly, a significant difference for the vertical chin parameter (CTV) was found between Class I and Class III, and between Class II and Class III.

**Conclusion:**

Both upper and lower lip thicknesses were highest for Class III followed by Class I and Class II, respectively. Lip lengths too were found to be highest for Class III skeletal relation. Nasolabial angle was larger in Class II malocclusion when compared to Class I and Class III. Similarly, both nasal length and nasal height measurements were in the order of Class III > Class II > Class I. Both horizontal and vertical chin parameters were larger for Class III sagittal relation.

## 1. Introduction

With the advent of more sophisticated diagnostic instruments and methods, orthodontic diagnosis and treatment planning have become more precise, and still not much of attention is paid toward soft tissue when compared to the hard tissues of the orofacial region.

A considerable body of evidence exists today, which counters the earlier concepts of constancy of soft tissue growth with their underlying hard tissue structures [1, 2].

In recent times, orthodontists are more concerned about facial esthetics. Analysis of both dental and skeletal patterns alone may prove inadequate or misleading, for marked variation exists in the soft tissue covering the dentoskeletal framework. Because soft tissue may vary in different persons in thickness, length, and postural tone, it is necessary to study directly the integumental contour of the face in order to consider facial harmony adequately [3]. A lot of information has come forward regarding what constitutes an attractive face [4, 5]. It has been found that the determinants for an esthetic face differ from region to region and from one ethnic group to another [6, 7]. Studies have been done regarding soft tissue profiles and dimensions of nose, lips, and chin in various part of the world [2, 8–10]. Study by Kamak and Celikoglu in Turkish orthodontic patients found significant differences in soft tissue thickness among different skeletal malocclusions for labrale superius, stomion, and labrale inferius sites in both male and female patients [9]. Study in adult Caucasian orthodontic patients from the mid-Balkan region showed that men had thicker facial soft tissue compared with female patients in Class I and Class II Division 2, whereas female patients in Class II Division 1 had thicker facial soft tissue of the mentolabial sulcus and chin. Men and women with a skeletal Class III malocclusion showed no significant difference in their facial soft tissue thickness [11]. Similar study done by Chhibber et al. found statistically significant difference at points rhinion, subnasale, labrale superius, labrale inferius, and labiomental in skeletal Class I. Differences were found at nasion, subnasale, labrale superius, and labrale inferius in skeletal Class II [12]. Study performed in Pakistani subjects by Jeelani et al. found significant differences in facial soft tissue thickness at glabella, labrale superius, stomion, and labiomental among different skeletal classes in male patients and at labrale superius, labrale inferius, labiomental, and pogonion in female patients [10]. CBCT (cone beam computed tomography) study on Syrian orthodontic patient by Hajeer et al. found significant differences in facial soft tissue thickness among three skeletal classes, although statistically significant differences were not detected for all of the measurements [13]. Since no such study has been done in Nepalese population, this study was undertaken to measure and compare the lips, nose, and chin parameters in different skeletal malocclusion groups in orthodontic patients at BPKIHS, Nepal.

### 1.1. Objectives

1. To assess the nasal morphology in Class I, Class II, and Class III skeletal patterns.
2. To evaluate the lip morphology in Class I, Class II, and Class III skeletal patterns.
3. To evaluate the chin morphology in Class I, Class II, and Class III skeletal patterns.
4. To find whether there is any statistically significant difference in the nose, lip, and chin morphology in these skeletal malocclusions.

## 2. Materials and Methods

A descriptive, cross-sectional study was conducted on 237 lateral cephalograms of patients, taken with the same machine with the same magnification, obtained from Department of Orthodontics BPKIHS, Nepal. Ethical clearance was obtained from the institutional Review Committee, BPKIHS [Code No: IRC/2125/021]. The sample size was estimated using the formula:

(1) $$\begin{array}{c} n=\frac{Z^{2}\sigma^{2}}{E^{2}}, \end{array}$$

where *n* is total number of samples.

(2) $$\begin{array}{c} Z=1.96,\\ \sigma =9.04,\\ E=2. \end{array}$$

When these values were substituted in the abovementioned formula, the sample size estimated was 78.5, which is close to 79. The total sample size of the study was 237 since three similar groups were studied.

### 2.1. Inclusion Criteria

1. Subjects visited to Department of Orthodontics, BPKIHS.
2. Subjects in the age group of 18–30 years.
3. Good quality radiographs were all soft and hard tissue landmarks were clearly visible.
4. The radiographs were selected according to their skeletal antero-posterior jaw relationship (Class I, Class II, or Class III). ANB angle was chosen to classify the sagittal skeletal malocclusion group as it is simple and an immediate evaluation method with all the required landmarks approximately placed on midsagittal plane increasing its reliability with both lateral cephalometry and three-dimensional (3D) analysis [14, 15].

Class I skeletal relationship was considered when ANB angle was 2 to 4°, Class II skeletal relationship was considered when ANB angle was >4°, and Class III skeletal relationship was considered when ANB angle was <2° [16–18].

### 2.2. Exclusion Criteria

1. Patients who have undergone orthodontic/orthopedic/orthognathic surgical treatment, splint therapy, dental prosthesis, and plastic surgery.
2. Patients with history of trauma particularly to the orofacial region.
3. Patients with temporomandibular joint disorders, medical conditions that would affect the growth of the mandible and maxilla, systemic syndromes, and craniofacial anomalies.
4. Patients with cleft lip, cleft palate, obvious/gross nasal, and chin deformity.

Lateral cephalograms were hand traced with a 2B pencil on 0.003″ mm matt acetate paper on a view box.

**Figure 1:**
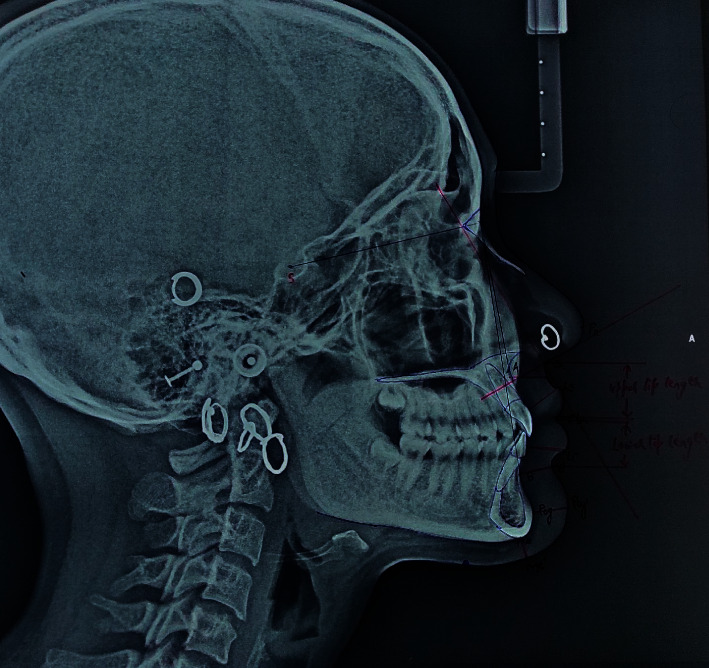
Cephalometric tracing used for the measurement of various soft tissue parameters.

The following vertical skeletal parameters were assessed (Figure 1):

1. SNA: angle formed by the intersection of Sella-Nasion and Nasion-A point.
2. SNB: angle formed by the intersection of Sella-Nasion and Nasion-B point.
3. ANB: angle formed by the intersection of Nasion-B point and Nasion-A point

The following soft tissue landmarks were identified to assess the nose (Figure 1):

1. Soft tissue nasion [N′]: the point of greatest concavity in the midline between the forehead and the nose.
2. Pronasale [Prn]: the tip of the nose [nasal tip].
3. Posterior columella point [PCm]: the most posterior point of the lower border of the nose at which it begins to turn inferiorly to merge with the philtrum of the upper lip.
4. Subnasale [Sn]: the deepest point at which the columella merges with the upper lip in the midsagittal plane.

The following nasal parameters were assessed (Figure 1):

1. Nose height (NH): Sn–N′—the distance between Sn (subnasale) and N′ (soft tissue N)
2. Nose length (NL): N′-Prn—the distance between N′ (soft tissue N) and Prn (pronasale)
3. Nasolabial angle [NLA]: the angle formed by the intersection of the PCm tangent and the PCm-Ls

The following soft tissue parameters were used to assess lips (Figure 1):

1. Labrale superius [Ls]: the point indicating the mucocutaneous border of the upper lip.
2. Labrale inferius [Li]: the median point in the lower margin of the lower membranous lip.
3. Stomion superius [Stms]: the lower most point on the vermillion of the upper lip.
4. Stomion inferius [Stmi]: the uppermost point on the vermillion of the lower lip.

The following lip parameters were assessed (Figure 1):

1. Basic upper lip thickness (BULT)—linear distance from 2 mm below A-point to subnasale.
2. Upper lip thickness (ULT)—linear distance from the most prominent labial point of the maxillary incisor [U1] to the labrale superius [Ls].
3. Basic lower lip thickness (BLLT)—linear distance from B-point to the deepest point of the labiomental fold.
4. Lower lip thickness (LLT)—linear distance from the most prominent labial point of the mandibular incisor [L1] to the labrale inferius [Li].
5. Upper lip length (ULL)—vertical distance from the subnasale to the lowest point on the upper lip [Stms] perpendicular to the F–H plane.
6. Lower lip length (LLL)—vertical distance from the highest point of the lower lip [Stmi] to the soft tissue B-point perpendicular to the F–H plane.

The following parameters were used to assess chin (Figure 1):

1. Pogonion (Pog): the most anterior point of the mandibular symphysis in the midline.
2. Menton (Me): the lowermost point of the mandibular symphysis in the midline.

The following soft tissue chin parameters were used for the study (Figure 1):

1. Chin thickness horizontal (CTH)—linear distance from the pogonion to its sagittal projection on the soft tissue.
2. Chin thickness vertical (CTV)—linear distance from menton to its vertical projection on the soft tissue.

## 3. Result

Two hundred and thirty-seven lateral cephalograms of orthodontics patients at Department of Orthodontics at BPKIHS were obtained and evaluated. 60 random samples were retraced and measured after an interval of 2 weeks. Data were entered and analyzed using Statistical Package for Social Sciences (version 16.0, SPSS Inc., Chicago, Illinois, USA). Intraclass correlation coefficient showed a high correlation between the two measurements, ranging from 0.922 for nasal length (NL) to 0.999 for ANB. Shapiro-Wilk test was used to determine the distribution of measurements of variables in each skeletal malocclusion group. It showed normal distribution of measurements with *p* > 0.05 for each individual measurement in different skeletal malocclusion groups. The descriptive statistics of mean and standard deviation was calculated for each variable. Multiple comparisons between groups were done with post hoc Bonferroni test. The average age of the patients was 23.33 years with standard deviation (SD) of 3.72 years.

As shown in Table 1, for lip parameters, basic upper lip thickness was found highest for Class III (13.26 ± 2.32 mm), followed by Class I (12.73 ± 2.57 mm) and Class II (12.67 ± 2.69 mm). Basic lower lip thickness (BULT) was greatest for Class II followed by Class III and Class I. Upper lip length (ULL) was in the order of Class II > Class III > Class I, whereas lower lip length (LLL) was in the order of Class III > Class I > Class II. Upper lip thickness (ULT) of 10.96 ± 3.03 mm was found in Class III. Class I had 10.7 ± 3.49 mm, and Class II had 9.59 ± 2.84 mm of ULT. For lower lip thickness (LLT), Class II showed highest mean thickness of 12.64 ± 3.25 mm followed by Class III (12.36 ± 2.86 mm) and Class I (12.17 ± 3.18 mm).

For nose variables, nasolabial angle (NLA) was greatest for skeletal Class II (98.66° ± 11.81°), this was followed by Class I (95.17° ± 12.86°), and least NLA was found in skeletal Class III (91.35° ± 14.7°). Both nasal length (NL) and nasal height (NH) were largest for Class III and least for Class I as shown in Table 2.

Table 3 shows the mean dimensions for chin variables. Both the horizontal (CTH) and vertical (CTV) chin parameters were the largest for Class III, followed by Class I and Class II.

On intergroup comparison for lip parameters (Table 4), significant differences were only found between Class II and Class III for upper lip thickness, and for lower lip thickness, between Class III and Class I, and between Class III and Class II.

The only significant difference seen on intergroup comparison of nasal parameters was between Class II and Class III for nasolabial angle (NLA) (Table 5).

Significant difference was only found for the vertical soft tissue chin (CTV) dimension. It was found between Class III and Class I, and between Class III and Class II (Table 6).

## 4. Discussion

In this economically, socially, and sexually competitive world, a pleasing appearance is a necessity [19]. Every one of us has different perceptions about what constitutes a beautiful face. Our own expressions, interpretation, and experiences influenced by culture and self-image make it unique.

Esthetic results, sometime, are more important to a patient than the proper alignment of teeth and occlusion. Hence, a good facial appearance along with good occlusion is the most important objective of orthodontic treatment. Since a proportionate or improvement of soft tissue profile does not necessarily accompany dentition changes, one can no longer rely entirely on dentoskeletal analysis for accurate information on the soft tissue facial profile [20].

The study comprised of 237 samples with 79 samples each in Class I, Class II, and Class III skeletal malocclusion groups. In each group, several parameters related to lip, chin, and nose were measured and analyzed.

Among the various parameters studied for upper lip morphology, BULT and ULT were found to be highest for Class III (13.26 ± 2.32; 10.96 ± 3.03) and lowest for Class II (12.67 ± 2.69; 9.59 ± 2.84). Since most of the skeletal Class III has maxillary hypoplasia, there is an increase in soft tissue volume to mask the degree of hypoplasia, resulting in a thick upper lip. However, the difference was statistically significant only for ULT between Class II and Class III. This is in agreement with study by Yan et al. [21], and they too found an increased upper lip thickness in Class III followed by Class I and Class II, and in partial agreement with the study of Dr. Neeraja [22]. In his study, he found significant differences between Class I and Class III, and between Class II and Class III. Safarzadeh et al. [23] compared the soft tissue thickness difference between male and female patients in various anterior-posterior skeletal classifications and found that soft tissue thickness at subnasale was highest for Class III followed by Class II and Class I for female patients. Similar study done by Mahto et al. [24] also found greater soft tissue thickness at subnasale for Class III compared with Class I and Class II. These results too are in agreement with the result of this study for BULT, whereas BLLT and LLT were highest for Class II (9.86 ± 1.77; 12.64 ± 3.25) and lowest for Class I malocclusion (9.66 ± 1.64; 12.17 ± 3.18). The difference found was not statistically significant. This is in agreement with the result of study by Safarzadeh et al. [23], for soft tissue thickness at labrale inferius.

For lip length, although the difference was not statistically significant, ULL was greatest for Class III (20.86 ± 3.36) and least for Class I (19.98 ± 4.17). LLL was greatest for Class III (17.99 ± 3.66) and least for Class II (15.09 ± 3.19), and the difference was statistically significant between Class III and Class I (*p*=0.001) and between Class III and Class II (*p* < 0.001).

Among the nasal parameters studied, NLA was greatest for Class II, followed by Class I and least for Class III. The difference was only statistically significant for Class II and Class III. This finding is in agreement with the study done by Arshad et al. [25] and Perović et al. [26]. They too found greatest NLA for Class II malocclusion followed by Class I and Class III, although the difference found was not statistically significant. However, the finding of this study is in partial agreement with the findings of the study by Asif et al. [27] where they too found the least nasolabial angle for Class III. However, it differs regarding the highest NLA. They found the highest NLA for Class I malocclusion. This is also in partial agreement with the study of Bhardwaj et al. [28] and Habib et al. [29]. They too found the highest NLA for Class I followed by Class II and least for Class III.

Class III samples had the greatest NL and Class I subjects had the least NL, although the findings were not statistically significant. This is in partial agreement with Bhardwaj et al. [28], where they found significant differences in NL of Class III subjects compared with Class I and Class II.

Class III samples had the greatest NH and Class I subjects had the least NH, although the findings were not statistically significant. This differs from the finding of Jafarpour et al. [30]. Nasal height was the least in Class III skeletal relation in their study. This difference may be due to racial and ethnic differences.

Both the horizontal (CTH) and vertical (CTV) chin parameters studied showed the highest mean value for Class III and lowest mean for Class II malocclusion group. Although no statistically significant difference was found for CTH, a statistically significant difference was found between Class III and Class I, and for Class III and Class II. This is in agreement with the findings of the study by Kurkcuoglu et al. [8] in Turkish female patients, but not for Turkish male patients. This result in chin morphology contrasts with the finding of Utsuno et al. [31], where they found highest horizontal chin dimension for Class II and the highest vertical chin dimension for skeletal Class I.

The present study suggests that there is racial and ethnic variation in soft tissue profile. The soft tissue profile also depends on the sagittal skeletal relation of the maxilla to the mandible, as the soft tissue tries to mask the skeletal discrepancies, and there is a difference in their thickness and angular measurement. Proper evaluation of the soft tissue morphology of an orthodontic patient is most especially when planning for growth modification and orthognathic surgery and can also help in forensic reconstruction of the face of an individual. However, the main objective of this study was to measure and compare the lips, nose, and chin parameters in different skeletal malocclusion groups in orthodontic patients.

## 5. Conclusion

The present study found that lip thickness, both upper and lower, was highest for Class III followed by Class I and least for Class II. Lip length too was found to be highest for Class III skeletal relation. As for the nasolabial angle, it was larger in case of Class II when compared to Class I and Class III. Similarly, both nasal length and nasal height measurements were in the order of Class III > Class II > Class I. Both horizontal and vertical chin parameters were larger for Class III sagittal relation.

## 6. Limitations

There are certain limitations to our study. First being the racial variation in soft tissue thickness as suggested by the literature. Since this study was done in rather localized population, the findings of this study cannot be generalized. Second, gender-based evaluation of soft tissue thickness was not done in this study because of disproportionate number of male and female patients. Third, since we used lateral cephalogram for the measurements, both sides of the face could not be evaluated. As studies have shown that there is unequal soft tissue thickness on right and left sides of the face, it would have been better to use three-dimensional radiograph for soft tissue evaluation.

## Figures and Tables

**Table 1:** Descriptive statistics of different variables among different skeletal malocclusion groups.

Variables	Class I, *N* = 79 mean (SD)	Class II, *N* = 79 mean (SD)	Class III, *N* = 79 mean (SD)	Total, *N* = 237 mean (SD)
BULT	12.74 (2.58)	12.67 (2.69)	13.26 (2.32)	12.89 (2.54)
BLLT	9.66 (1.65)	9.87 (1.78)	9.70 (1.73)	9.74 (1.71)
ULL	19.99 (4.17)	20.87 (3.36)	20.57 (4.13)	20.47 (3.91)
ULT	10.71 (3.50)	9.59 (2.85)	10.97 (3.04)	10.42 (3.18)
LLT	12.18 (3.18)	12.64 (3.26)	12.37 (2.86)	12.40 (3.10)
LLL	16.00 (3.59)	15.10 (3.19)	17.99 (3.67)	16.36 (3.68)

**Table 2:** Mean values of nose variables among different skeletal malocclusion groups.

Variables	Class I, *N* = 79 mean (SD)	Class II, *N* = 79 mean (SD)	Class III, *N* = 79 mean (SD)	Total, *N* = 237 mean (SD)
NLA	95.18 (12.87)	98.67 (11.81)	91.35 (14.71)	95.06 (13.46)
NL	44.78 (7.45)	46.27 (7.27)	47.13 (7.50)	46.06 (7.44)
NH	49.28 (8.12)	50.42 (8.64)	51.92 (8.02)	50.54 (8.30)

**Table 3:** Mean values of chin variables among different skeletal malocclusion groups.

Variables	Class I, *N* = 79 mean (SD)	Class II, *N* = 79 mean (SD)	Class III, *N* = 79 mean (SD)	Total, *N* = 237 mean (SD)
CTH	9.24 (2.53)	9.21 (2.43)	9.52 (2.93)	9.32 (2.63)
CTV	6.05 (1.62)	5.81 (1.85)	6.89 (2.55)	6.25 (2.09)

**Table 4:** Intergroup comparison of lip variables among different sagittal skeletal groups.

Variables	Class I—Class II mean difference (confidence interval) *p*	Class I—Class III mean difference (confidence interval) *p*	Class II—Class III mean difference (confidence interval) *p*
BULT	0.06 (−0.91 to 1.04) 1.00	−0.53 (−1.50 to 0.44) 0.577	−0.59 (−1.56 to 0.38) 0.431
BLLT	−0.20 (−0.86 to 0.46) 1.00	−0.04 (−0.70 to 0.62) 1.00	0.16 (−0.50 to 0.82) 1.00
ULL	−0.88 (−2.38 to 0.62) 0.475	−0.58 (−2.08 to 0.92) 1.00	0.30 (−1.20 to 1.80) 1.00
ULT	1.11 (−0.09 to 2.32) 0.081	−0.26 (−1.47 to 0.94) 1.00	−1.37 (−2.58 to −0.17) 0.019^*∗*^
LLT	−0.47 (−1.66 to 0.72) 1.00	−0.19 (−1.38 to 1.00) 1.00	0.28 (−0.91 to 1.47) 1.00
LLL	0.90 (−0.43 to 2.24) 0.315	−1.99 (−3.33 to −0.65) 0.001^*∗*^	−2.89 (−4.23 to −1.55) <0.001^*∗*^

^*∗*^Mean difference is significant at *p* < 0.05.

**Table 5:** Intergroup comparison of nose variables among different sagittal skeletal groups.

Variables	Class I–Class II mean difference (confidence interval) *p*	Class I–Class III mean difference (confidence interval) *p*	Class II–Class III mean difference (confidence interval) *p*
NLA	−3.49 (−8.54 to 1.57) 0.294	3.83 (−1.23 to 8.89) 0.208	7.31 (2.26 to 12.37) **0.002**^*∗*^
NL	−1.49 (−4.33 to 1.35) 0.623	−2.35 (−5.19 to 0.49) 0.143	−0.86 (−3.70 to 1.98) 1.00
NH	−1.13 (−4.30 to 2.03) 1.00	−2.64 (−5.81 to 0.53) 0.137	−1.50 (−4.68 to 1.66)0.76

^*∗*^Mean difference is significant at *p* < 0.05.

**Table 6:** Intergroup comparison of chin variables among different sagittal skeletal groups.

Variables	Class I–Class II mean difference (confidence interval) *p*	Class I–Class III mean difference (confidence interval) *p*	Class II–Class III mean difference (confidence interval) *p*
CTH	0.03 (−0.98 to 1.04) 1.00	−0.29 (−1.30 to 0.72) 1.00	−0.32 (−1.33 to 0.69) 1.00
CTV	0.25 (−0.54 to 1.03) 1.00	−0.84 (−1.63 to −0.05) **0.031**^*∗*^	−1.09 (−1.87 to −0.30) **0.003**^*∗*^

^*∗*^Mean difference is significant at *p* < 0.05.

## Data Availability

The data used in this study can be obtained from the corresponding author upon request.
